# Effects of Digeda-4 Decoction on the CYP450 Activities in Rats Using a Cocktail Method by HPLC

**DOI:** 10.1155/2018/1415082

**Published:** 2018-10-23

**Authors:** Dong Zhang, Guo-dong Wu, Yan-dong Zhang, Jian-ping Xu, Hai-tao Zhen, Min-hui Li

**Affiliations:** ^1^Baotou Medical College, Baotou 014060, China; ^2^Inner Mongolia Institute of Traditional Chinese Medicine, Hohhot 010020, China

## Abstract

Digeda-4 decoction is a traditional Mongolian medicine; its effects on cytochrome (CYP) enzymes are still unclear. CYP450 isoenzymes are the main drug metabolic enzymes, and their activities may be induced or inhibited by certain drugs, which lead to drug interactions in clinical use. Effects of Digeda-4 decoction on the activities of CYP450 subtype enzymes CYP1A2, CYP2C9, CYP2E1, CYP2C19, and CYP3A4 in rats were studied by cocktail method, and the pharmacokinetic parameters of five specific probe drugs (theophylline, tolbutamide, chlorzoxazone, omeprazole, and midazolam) were calculated by DAS software; changes of parameters can be used to evaluate the effects of Digeda-4 decoction on enzyme activities. The experimental rats were divided into three groups: control group, Digeda group, and positive group. Rats in Digeda group were given Digeda-4 decoction through continuous gavage for 14 days. After fasting for 12 hours, the mixed probes drug solution was injected into the tail vein; the blood samples were collected through the orbital vein at different time points. The concentrations of probe drugs in rat plasma were measured by HPLC. Compared with the control group, the half-life time (t_1/2_) of the pharmacokinetic parameters of theophylline, tolbutamide, omeprazole, and midazolam was prolonged, the area under the curve (AUC) increased, and the plasma clearance (CL) decreased in the Digeda group. Continuous gavage administration for 14 days may inhibit the activities of CYP450 subtype enzymes CYP1A2, CYP2C9, CYP2C19, and CYP3A4 of rats. Herb-drug interaction should be noted between Digeda-4 decoction and the drugs metabolized by CYP1A2, CYP2C9, CYP2C19, and CYP3A4.

## 1. Introduction

Traditional herbs are widely used throughout the world, while the herb-drug interactions resulting from which are increasingly reported. For example,* Hypericum perforatum* is an inducer of cytochrome (CYP) 3A4 and can also inhibit or induce other subtype enzymes.* H. perforatum* could reduce blood levels of indinavir, warfarin, digoxin, amitriptyline, midazolam, cyclosporine, and so forth [1]. There are several cases reporting that* H. perforatum* reduced the blood concentration of cyclosporine, resulting in organ rejection, such as the case of a kidney transplant patient who had been taking cyclosporin for several years in whom organ rejection appeared after taking* H. perforatum*. After the challenge test, the same result was obtained. The blood concentration of cyclosporin decreased to 0.48 ng/(mL x mg) from 0.84 ng/(mL x mg) after combination with* H. perforatum*; the herb-drug interaction was the cause of organ rejection [2].* H. perforatum* also caused breakthrough bleeding in women who take steroids-containing contraceptive pills [3].* Ginseng* is found in Eastern Asia and North America. The roots and leaves of* Ginseng* can be used to make pills, coffee drinks, energy drinks, and tea. A patient who took imatinib for 7 years suffered liver damage after taking* Ginseng *as an energy drink for 3 months, which may have been caused by interaction between imatinib and* Ginseng* [4]. This interaction may be due to the active constituents in* Ginseng, *which influenced the expression of the CYP450 enzymes [5]. Numerous studies indicate that herbal medicines can affect the metabolism of drugs' combined use with them by inducing or inhibiting drug-metabolizing enzymes, which pose a great risk. Therefore, it is urgent to study the effects of herbs on CYP450 enzymes and to guide the clinical application of herbs in a better way.

“Digeda-4 decoction” (alias of Leizhuhua-4 decoction) is a traditional Mongolian medicine, which is composed of Lomatognii Herba (*Lomatogonium rotatum* Fries ex Nym.), Picrorrhizae Rhizoma (*Picrorhiza scrophulariiflora* Pennell), Gardeniae Fructus (*Gardenia jasminoides* Ellis), and Dianthi Herba (*Dianthus superbus* L.); the proportion of the four herbs is 1:1:1:1. It cames from Zhen Zhi Ming Yi Dian and is included in the pharmaceutical standards of the ministry of health of the People's Republic of China and Mongolian Drug Standards Booklet 1998 [6].* Lomatogonium rotatum* is cultivated mainly in East-Central Inner Mongolia, including Hexigten banner, Duolun county, Zhenglan banner, and so forth [7]. This plant mainly contains compounds such as flavonoids, triterpenoids, iridoids, and cleaved iridoids [8].* Picrorhiza scrophulariiflora* is mainly distributed in northwest of Yunnan province and the alpine meadows and stone heaps of Tibet [9]. The plant contains a large amount of iridoid glycosides, phenylethanoid glycosides, phenol glycosides, cucurbitane triterpenoids, and a very small amount of flavonoids and aromatic acids [10]. The authentic production areas of Gardenia jasminoides are Jiangxi, Qingjiang, Fujian, and Hubei [11]. Its chemical compositions include iridoids, iridoid glucosides, triterpenoids, organic acids, and volatile compounds, among which geniposide, genipin, gardenoside, and crocin are the major bioactive compounds [12].* Dianthus superbus* is distributed throughout Inner Mongolia, Liaoning, Hebei, Henan, Hubei, Jiangsu, and Zhejiang; the main cultivated areas are the east and southeast of Daxing anling forest region [13, 14]. It contains triterpenoid saponins, flavones, cyclopeptides, frangulic acid, and polysaccharide [14–16]. Some scholars have studied that Digeda-4 decoction contains more swertiamarin and also contains geniposide, picrosides II, and total flavonoids of Dianthus superbus [17]. This decoction is beneficial to liver and cholecyst, often combined with other Chinese and Mongolian prescriptions or western medicines in clinical application [18]. Modern pharmacological studies have shown that Digeda-4 decoction has a protective effect on chemical liver injury and has a regulatory effect on the lipid metabolism disorder of rats caused by carbon tetrachloride [19, 20]. However, whether Digeda-4 decoction can affect the metabolic process of its coadministered drugs by influencing metabolic enzymes is still unknown.

CYP450 is a family of heme proteins that are involved in the metabolism of endogenous and exogenous substances [21]. This study involved five important CYP isozymes, CYP1A2, CYP2C9, CYP2E1, CYP2C19, and CYP3A4. These enzymes were chosen because they account for a high proportion of total CYPs (CYP3A accounts for approximately 30%, CYP2C accounts for approximately 20%, CYP1A2 accounts for approximately 13%, and CYP2E1 accounts for approximately 7%) and participate in the metabolism of a considerable number of drugs [22]. These enzymes can be induced or inhibited by other exogenous substances. In clinical practice, inducing or inhibiting the activity of CYP450 enzymes may affect the pharmacokinetics of the drug, leading to accidental or even serious clinical drug-drug interactions (DDIs) [23, 24]. Enzyme inhibition by inhibitor drugs could lead to concentration increase of another drug in plasma and increase the risk of adverse drug reactions (ADRs) or exert toxic effects [25]. It is important to study the induction or inhibition of CYP450 enzyme activity in order to predict the potential DDIs, thereby to avoid the occurrence of adverse reactions. The cocktail approach has become one of the basic analytical tools to evaluate DDIs* in vivo*. Compounds specifically catalyzed by each CYP isoform, known as probe drugs, have been widely used to assess the activity of various CYP450s in this approach [26].

To evaluate the safety of clinical combination use, the influences of Digeda-4 decoction on the activities of five CYP450 enzymes in rats are evaluated according to the pharmacokinetic parameters' changes using five specific probe drugs (theophylline for CYP1A2, tolbutamide for CYP2C9, chlorzoxazone for CYP2E1, omeprazole for CYP2C19, and midazolam for CYP3A4). Two sensitive and specific HPLC methods were used to quantify the concentration of five probe drugs.

## 2. Materials and Methods

### 2.1. Chemicals and Reagents

Digeda-4 decoction was purchased from the pharmacy department of Mongolian traditional Chinese hospital (Baotou, Inner Mongolia, China). Theophylline, tolbutamide, chlorzoxazone, midazolam, omeprazole, (all > 98%), and the internal standard (IS) tinidazole were purchased from Dalian Meilun Biotech Co., Ltd. (Dalian, China). HPLC-grade acetonitrile and methanol were from Merck Company (Darmstadt, Germany). All other chemicals were of analytical grade. Ultrapure water (resistance > 18 mΩ) was prepared using a Millipore Milli-Q purification system (Bedford, USA).

### 2.2. Animals

Wistar rats (male, 220 ± 20 g) were purchased from Beijing Sibefu Biotechnology Co., Ltd. Animals were housed under natural light-dark cycle conditions with controlled temperature (22°C). All rats were housed at Laboratory Animal Research Center of Baotou Medical College. All experimental procedures were approved ethically by the Administration Committee of Experimental Animals of Baotou Medical College.

### 2.3. Instrumentation and Conditions

UltiMate3000 HPLC equipped with diode array detector was used to analyze the compounds. Theophylline, tolbutamide, chlorzoxazone, omeprazole, midazolam, and tinidazole (IS) were separated using Eclipse XDB-C18 (4.6 × 150 mm, 5 *μ*m, Agilent, USA) maintained at 35°C. The initial mobile phase consisted of acetonitrile and water (containing 0.1% phosphoric acid) with gradient elution at a flow rate of 1.0 mL/min and an injection volume of 10 *μ*L. Elution was in a linear gradient, condition A: with the acetonitrile content changing from 8% to 50% between 0 and 30 min for theophylline, tolbutamide, chlorzoxazone, and IS, and condition B: with the acetonitrile content changing from 10% to 40% between 0 and 14min for omeprazole, midazolam, and IS.

### 2.4. Preparation of Standard Solutions

Stock solutions of 1.0 mg/mL each of theophylline, tolbutamide, chlorzoxazone, omeprazole, midazolam, and IS were prepared by methanol. The working standard solutions of each analyte were prepared by serial dilution of the stock solution with methanol. All the solutions were stored at 4°C and brought to room temperature before use.

The calibration standards were prepared by spiking blank rat plasma with appropriate amounts of theophylline, tolbutamide, chlorzoxazone, omeprazole, and midazolam. Calibration plots of theophylline, tolbutamide, and chlorzoxazone were constructed in the range of 0.1-100 *μ*g/mL for plasma (100, 50, 25, 10, 5, 2.5, 1, 0.5, 0.25, and 0.1 *μ*g/mL). Calibration plots of omeprazole and midazolam were constructed in the range of 0.1-50 *μ*g/mL for plasma (50, 25, 10, 5, 2.5, 1, 0.5, 0.25, and 0.1 *μ*g/mL).

### 2.5. Pharmacokinetic Study

Forty-eight male Wistar rats (220 ± 20 g) were randomly divided into two control groups, two Digeda groups, and two positive groups (n=8). Control groups and Digeda groups were given saline and Digeda-4 decoction (600 mg/kg), respectively, by continuous intragastric administration for 14 days, while positive groups were given phenobarbital injection (50 mg/kg) by continuous intraperitoneal injection for 7 days. After the last dose, one control group, one Digeda group, and one positive group were all given the mixture of three probe drugs (theophylline, tolbutamide, and chlorzoxazone were 10 mg/kg, 2.5 mg/kg, and 10 mg/kg) by intravenous injection. The other groups were given the mixture of two probe drugs (omeprazole and midazolam were 10 mg/kg and 5 mg/kg) by intravenous injection.

Blood samples (0.8 mL) were collected from the orbital vein at 0.08, 0.16, 0.25, 0.33, 0.5, 1, 2, 4, 6, 8, 12, and 24 hours after intravenous injection of probe drugs (theophylline, tolbutamide, and chlorzoxazone) and 0.08, 0.16, 0.25, 0.33, 0.5, 1, and 2 hours after intravenous injection of probe drugs (omeprazole and midazolam). The samples were immediately centrifuged at 3500 r/min for 10 min, and 200 *μ*L plasma was obtained from each sample.

The plasma samples were extracted. In a 5 mL centrifuge tube, 100 *μ*L of the IS working solution (0.1 mg/mL) was added to 200 uL of collected plasma sample followed by the addition of 2 mL of dichloromethane. After the tube was vortex-mixed for 5 min, the sample was centrifuged at 3500 r/min for 10 min. 1.2 mL of the organic phase was transferred into the other glass tube and was dried by nitrogen. The residue was redissolved with 200 *μ*L methanol and then injected into the HPLC system for analysis. The concentration of probe drugs in plasma was measured by HPLC.

### 2.6. Statistical Analysis

Plasma probe drug concentration versus time data for each rat was analyzed by DAS software (version 3.0, Drug Clinical Research Center of Shanghai University of TCM and Shanghai BioGuider Medicinal Technology, Co. Ltd., China). The pharmacokinetic parameters of the Digeda group and control group probe drugs with the* t*-test were analyzed using SPSS 17.0 statistical software. p < 0.05 was considered statistically significant.

## 3. Results

### 3.1. Method Validation

#### 3.1.1. HPLC Chromatograms

**Figure 1:**
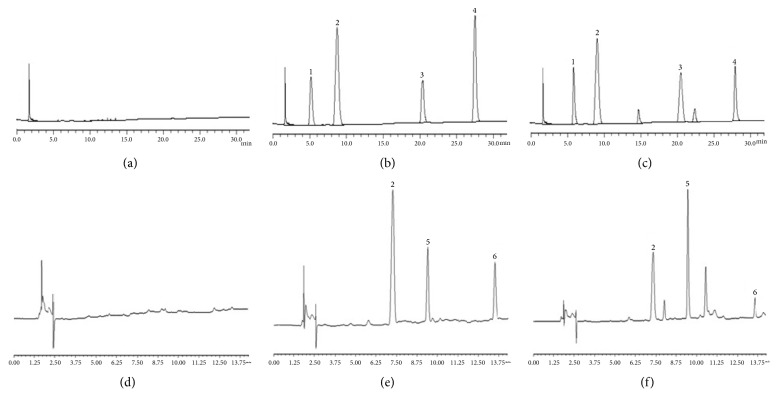
HPLC chromatograms. ((a) and (d)) Blank plasma. ((b) and (e)) Blank plasma spiked with the cocktail probe drugs and the IS. ((c) and (f)) Plasma sample obtained from a rat after intravenous injection of the cocktail probe drugs spiked with the IS; 1, theophylline; 2, tinidazole; 3, chlorzoxazone; 4, tolbutamide; 5, omeprazole; 6, midazolam.

Figure 1 showed HPLC chromatograms for the probe drugs. In condition A, the retention time was 5.192 min (theophylline), 8.930 min (tinidazole), 20.374 min (chlorzoxazone), and 27.512 min (tolbutamide); and in condition B, it was 7.098 min (tinidazole), 9.208 min (omeprazole), and 13.153 min (midazolam). They had a better separation effect.

#### 3.1.2. Calibration Curve and Quantitative Limit

The linear regressions of the peak area ratios versus concentrations were fitted over the concentration range of 0.1-100 *μ*g/mL (for theophylline, tolbutamide, and chlorzoxazone) and 0.1-50 *μ*g/mL (for midazolam and omeprazole) in rat plasma. Typical equations of the calibration curves are listed in Table 1. Each probe drug peak area ratio with concentration has a good linear relationship in the range of concentration. The LOQ for probe drugs in plasma was 0.1 *μ*g/mL.

#### 3.1.3. Precision and Extraction Efficiency

Intraday precision was measured to be 8.33% or less and the interday precision was measured to be 9.63% or less at each level. Mean extraction efficiency was measured to be 86.59-111.41%. Assay performance data are presented in Table 2.

#### 3.1.4. Stability

The results of room temperature, freeze-thaw, and long-term (20 days) stability indicated that the analyte was stable under various storage conditions, since the bias in concentration was within ±10% of their nominal values.

### 3.2. Effects of Digeda-4 Decoction on CYP450 Activities in Rats

**Figure 2:**
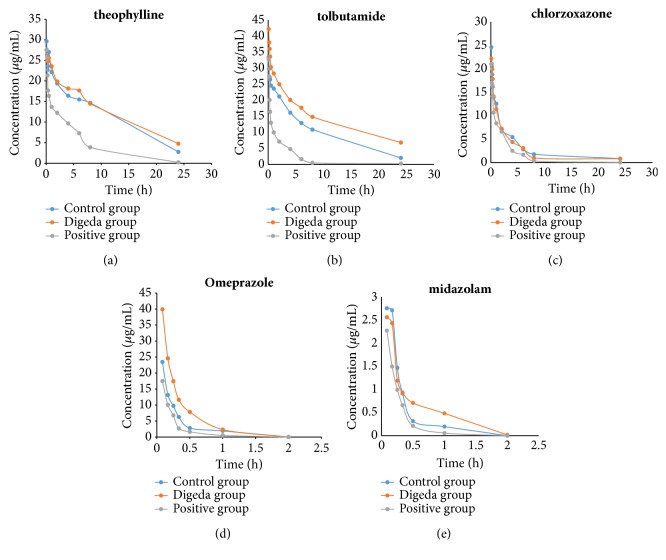
Mean plasma concentration-time curves of theophylline (a), tolbutamide (b), chlorzoxazone (c), omeprazole (d), and midazolam (e).

This method was applied to the pharmacokinetic study of five probe drugs in rats. The mean plasma concentration-time curves are shown in Figure 2. The main pharmacokinetic parameters after intravenous injection of theophylline, tolbutamide, chlorzoxazone, omeprazole, and midazolam from noncompartment model analysis are summarized in Table 3. According to the experiment in the Digeda and control groups, there was a statistically significant difference in the half-life (t_1/2_), area under the curve (AUC), and the plasma clearance (CL) for theophylline, tolbutamide, omeprazole, and midazolam (p < 0.05 or p < 0.01), while there was no statistical difference in chlorzoxazone (p > 0.05). Digeda-4 decoction may affect the activity of CYP1A2, CYP2C9, CYP2C19, and CYP3A4 in rats, yet it may not affect the activity of CYP2E1 in rats.

## 4. Discussion

CYP1, CYP2, and CYP3 were major CYP450 isoforms and participate in approximately 75% of different metabolic responses [27]. CYP1A2 plays an important role in the metabolism of several commonly used drugs, such as cardiovascular drugs (triamterene, guanabenz, and propranolol), antipyretic analgesic anti-inflammatory drugs (acetaminophen, nabumetone, and phenacetin), local anesthetics, antiarrhythmic drugs (lidocaine), antidepressant (duloxetine), muscle relaxant (tizanidine), antipsychotics (clozapine and olanzapine), hypnotic drug (zolpidem), and cholinesterase inhibitor (tacrine) [28, 29]. Some endogenous substances or exogenous drugs require biological activation by CYP1A2, such as endogenous substrate estrogen, melatonin, arachidonic acid, and so forth, as well as exogenous antiandrogen drug flutamide [30]. CYP2C9 may participate in metabolism of 16% clinical drugs, such as AT_1_ receptor blockers losartan and candesartan, oral hypoglycemic drugs tolbutamide and glibenclamide, the anticonvulsants phenytoin and valproic acid, the anticoagulant warfarin, and most NSAIDs: ibuprofen, celecoxib, diclofenac, and meloxicam; endogenous substances such as arachidonic acid and some steroids are also metabolized by CYP2C9 [31–35]. Progesterone and melatonin were the endogenous substrates of CYP2C19 [29]. CYP2C19 was the main enzyme of metabolic proton pump inhibitors (including omeprazole and pantoprazole) and antidepressant citalopram and also the main enzyme for the metabolic activation of the anticoagulant clopidogrel [36–39]. CYP2E1 plays a vital role in the metabolism of alcohol, organic solvents drugs (such as halothane, chlorzoxazone, and paracetamol), toxin, lipid, carcinogen, and procarcinogens [27, 40, 41]. Besides, 40%-50% of clinically used drugs are metabolized by CYP3A4, such as macrolide antibiotics, calcium channel blockers, statins (atorvastatin, lovastatin, and simvastatin), and anticoagulants coagulation factor Xa inhibitor (apixaban and rivaroxaban) [42].

As shown in Table 3, compared with the control group, the Digeda group had longer t_1/2_, increased AUC, and decreased CL, these pharmacokinetic parameters were significantly different in theophylline, tolbutamide, omeprazole, and midazolam; in Figure 2, the AUC of the theophylline, tolbutamide, omeprazole, and midazolam in the Digeda group were also higher than those in the control group. The information in Table 3 and Figure 2 can be obtained that Digeda-4 decoction inhibited CYP1A2, CYP2C9, CYP2C19, and CYP3A4 enzymes in rats. These results indicated that herb-drug interactions may occur between Digeda-4 decoction and drugs metabolized by CYP1A2, CYP2C9, CYP2C19, and CYP3A4 enzymes, probably due to the inhibitory effect of Digeda-4 decoction on CYPs. This inhibition of the enzyme may result in an increase in plasma concentration when the drugs mentioned above were combined with Digeda-4 decoction, which will bring the risk of adverse effects, even toxic reaction. Therefore, in clinical practice, Digeda-4 decoction should be carefully used when it is associated with drugs metabolized by CYP1A2, CYP2C9, CYP2C19, and CYP3A4 enzymes; if the association is required, the drug delivery plan needs to be adjusted to avoid overdosage or high concentration. Table 3 showed that, in the experiment of Digeda group and control group, there was insignificant difference in pharmacokinetic behaviors compared with the control group for chlorzoxazone, Figure 2 indicated that the AUC of chlorzoxazone in Digeda group was similar to the control group, indicating that the Digeda-4 decoction had no effect on the activity of CYP2E1 enzyme in rats. There was a potential guidance on clinical drug combination that Digeda-4 decoction could be considered as a safety combination drug with CYP2E1 metabolism drugs in clinical practice, while Digeda-4 decoction could not affect the metabolism of some small molecules such as carcinogen and procarcinogens.

Digeda-4 decoction consisted of four medicinal herbs, which were Lomatognii Herba, Picrorrhizae Rhizoma, Gardeniae Fructus, and Dianthi Herba. The main components of Lomatognii Herba were oleanolic acid and swertiamarin. Oleanolic acid can inhibit CYP1A2 activity significantly, but it has little inhibition on CYP2E1 activity and has no effect on CYP3A4 in healthy volunteers [43]; swertiamarin shows inhibitory effect on CYP2C9 activity in rat liver microsomes* in vitro* [44]. Gardeniae Fructus contains geniposide, which belongs to iridoid glycosides and can be used as a food-coloring agent and in the treatment of liver and inflammatory diseases. According to Han's research, geniposide has no inhibitory effect on CYP1A2, CYP2C9, CYP2C19, and CYP3A4 [45]. Picrorrhizae Rhizoma is used for the treatment of various liver and inflammatory conditions, the main active ingredients of which were Picrosides I and II [46]. According to Xiao's report, picroside II has inhibitory effect on the activity of CYP2C19 in* vivo* [47]. Dianthi Herba is widely used to treat urethritis, carbuncles, and carcinoma; it contained an amount of flavonoids [48]. There was no study of the effects of this drug on the CYP450. In conclusion, the inhibitory effect of Digeda-4 decoction on CYP1A2 may be related to the main component of Lomatognii Herba, oleanolic acid; the inhibitory effect on CYP2C9 may be related to swertiamarin; and the inhibitory effect on CYP2C19 may be related to the main component of Picrorrhizae Rhizoma, picroside II.

The results of* in vitro* and* in vivo* experiments may be different due to various factors; in general,* in vivo* experiments are more persuasive. This experiment mainly studied the effect of the whole formula on the enzyme activity* in vivo*, because formula was the main form of clinical application. The formula was composed of several kinds of medicinal materials, and they provided synergistic effects. The changes in enzyme activity may be related to any herb or any monomer compound or the combination of several herbs or the metabolic products. Further research needs to refer the reasons why the activities of CYP450 enzymes are inhibited. And whether Digeda-4 decoction affects the metabolism of drugs through other pathays or not is still pending.

## 5. Conclusion

In this experiment, the concentration of five probe drugs in rat plasma was successfully determined by HPLC, and the effect of Digeda-4 decoction on the activity of five CYP450 isoforms was evaluated. The results indicated that repeated oral administration of Digeda-4 decoction for 14 days at doses of 600 mg/kg/day inhibited the activity of CYP1A2, CYP2C9, CYP2C19, and CYP3A4 and had no effect on CYP2E1 in rats. Special attention should be paid to herb-drug interactions between Digeda-4 decoction and drugs metabolized by CYP1A2, CYP2C9, CYP2C19, and CYP3A4 enzymes. These results provided valuable information on the interactions of Digeda-4 decoction with some other drugs.

## Figures and Tables

**Table 1:** Regression equation and correlation coefficient for five probe drugs (y = peak area ratio of probe drugs vs IS; x = concentration of probe drugs).

Probe drugs	Regression equation	Correlation coefficient	Linear range (*μ*g/mL)
Theophylline	Y=0.0057x+0.0013	0.9994	0.1-100
Tolbutamide	Y=0.1938x+0.0004	0.9990	0.1-100
Chlorzoxazone	Y=0.0057x+0.0002	0.9999	0.1-100
Omeprazole	Y=0.0102x+0.0189	0.9986	0.1-50
Midazolam	Y=0.056x+0.0274	0.9994	0.1-50

**Table 2:** Precision and extraction efficiency of five probe drugs in rat plasma (n=5).

Compound	Concentration (*μ*g/mL)	Intraday precision		Interday precision		Extraction efficiency	RSD%
		x ± s	RSD%	x ± s	RSD%		
Theophylline	5	5.23±0.40	7.65	5.19±0.50	9.63	103.14±8.91	8.64
	25	23.31±1.35	5.79	22.74±1.79	7.87	92.08±6.09	6.61
	100	102.28±4.65	4.55	103.21±4.64	4.50	102.72±4.62	4.50
Tolbutamide	5	4.79±0.30	6.26	4.77±0.40	8.39	96.10±6.63	6.90
	25	24.73±1.31	5.30	26.10±1.49	5.70	101.65±5.79	5.70
	100	101.37±5.27	5.20	98.52 ±6.60	6.70	99.93±5.90	5.90
Chlorzoxazone	5	5.21±0.40	7.67	4.87±0.30	6.16	100.62±6.84	6.80
	25	25.23±1.08	4.28	24.81±1.19	4.80	99.73±4.49	4.50
	100	97.84±3.42	3.50	97.76±4.10	4.19	97.84±3.83	3.91
Omeprazole	0.5	0.52±0.03	5.77	0.47±0.02	4.26	95.33±5.15	5.40
	5	4.48±0.29	6.47	4.31±0.26	6.03	86.59±5.28	6.10
	20	18.79±1.33	7.07	18.86±1.28	6.79	95.13±6.35	6.67
Midazolam	0.5	0.48±0.04	8.33	0.49 ±0.04	8.16	94.53±8.12	8.59
	5	5.58±0.23	4.12	5.63±0. 17	3.02	111.41±2.95	2.65
	20	21.87±1.56	7.13	22.30±1.72	7.71	111.39±9.82	8.82

**Table 3:** Pharmacokinetic parameters of five probe drugs (mean±SD, n=8).

Probe drugs	Parameters	Control group	Digeda group	Positive group
Theophylline	T_1/2_/h	6.706±1.625	13.917±2.664*∗∗*	2.475±0.358
	Cmax/ug.mL^−1^	31.833±5.611	30.737±5.451	30.025±5.183
	AUC_0~t_/ug.mL^−1^.h	205.724±37.141	299.091±67.017*∗*	71.926±15.443
	AUC_0~*∞*_/ug.mL^−1^.h	325.588±76.856	568.825±141.842*∗∗*	117.825±21.367
	Cl/L.h^−1^.Kg^−1^	0.033±0.006	0.020±0.003*∗∗*	0.061±0.012
Tolbutamide	T_1/2_/h	6.055±1.081	14.947±3.336*∗*	4.812±0.930
	Cmax/ug.mL^−1^	35.642±4.106	40.204±6.834	33.572±3.967
	AUC_0~t_/ug.mL^−1^.h	165.361±31.618	253.703±59.841*∗*	66.621±15.387
	AUC_0~*∞*_/ug.mL^−1^.h	281.315±68.167	541.077±103.628*∗∗*	93.458±22.481
	Cl/L.h^−1^.Kg^−1^	0.011±0.002	0.005±0.001*∗∗*	0.021±0.004
Chlorzoxazone	T_1/2_/h	3.259±0.455	4.008±0.617	2.722±0.516
	Cmax/ug.mL^−1^	24.599±2.017	23.246±4.722	22.677±3.294
	AUC0~t/ug.mL^−1^.h	49.731±9.292	54.010±9.468	38.600±8.152
	AUC0~∞/ug.mL^−1^.h	56.041±10.531	65.272±10.513	42.349±9.768
	Cl/L.h^−1^.Kg^−1^	0.053±0.010	0.041±0.005	0.065±0.018
Omeprazole	T_1/2_/h	0.163±0.032	0.282±0.046*∗∗*	0.118±0.020
	Cmax/ug.mL^−1^	20.856±3.706	38.558±6.688*∗∗*	16.913±2.631
	AUC_0~t_/ug.mL^−1^.h	6.923±1.387	13.273±2.634*∗∗*	4.978±0.845
	AUC_0~*∞*_/ug.mL^−1^.h	7.283±1.311	14.153±2.609*∗∗*	5.344±0.993
	Cl/L.h^−1^.Kg^−1^	0.480±0.089	0.294±0.054*∗∗*	0.615±0.086
Midazolam	T_1/2_/h	0.144±0.030	0.199±0.032*∗*	0.105±0.016
	Cmax/ug.mL^−1^	2.900±0.491	2.675±0.509	2.556±0.507
	AUC_0~t_/ug.mL^−1^.h	0.881±0.151	1.166±0.209*∗*	0.653±0.095
	AUC_0~*∞*_/ug.mL^−1^.h	0.928±0.157	1.355±0.309*∗*	0.711±0.125
	Cl/L.h^−1^.Kg^−1^	6.230±1.145	4.183±0.743*∗∗*	6.896±0.964

Comparing Digeda group with the control group, t_1/2_, half-life time; Cmax, maximum plasma concentration; AUC, the area under the plasma concentration-time curve; CL, plasma clearance.

*∗* p < 0.05, *∗∗* p < 0.01

## Data Availability

The data used to support the findings of this study are available from the corresponding author.
